# Supplementary figures and images for: Interplay between the EMT transcription factors ZEB1 and ZEB2 regulates hematopoietic stem and progenitor cell differentiation and hematopoietic lineage fidelity
Source: PLoS Biol. 2021 Sep 22;19(9):e3001394. doi: 10.1371/journal.pbio.3001394 (PMC8489726; doi:10.1371/journal.pbio.3001394)

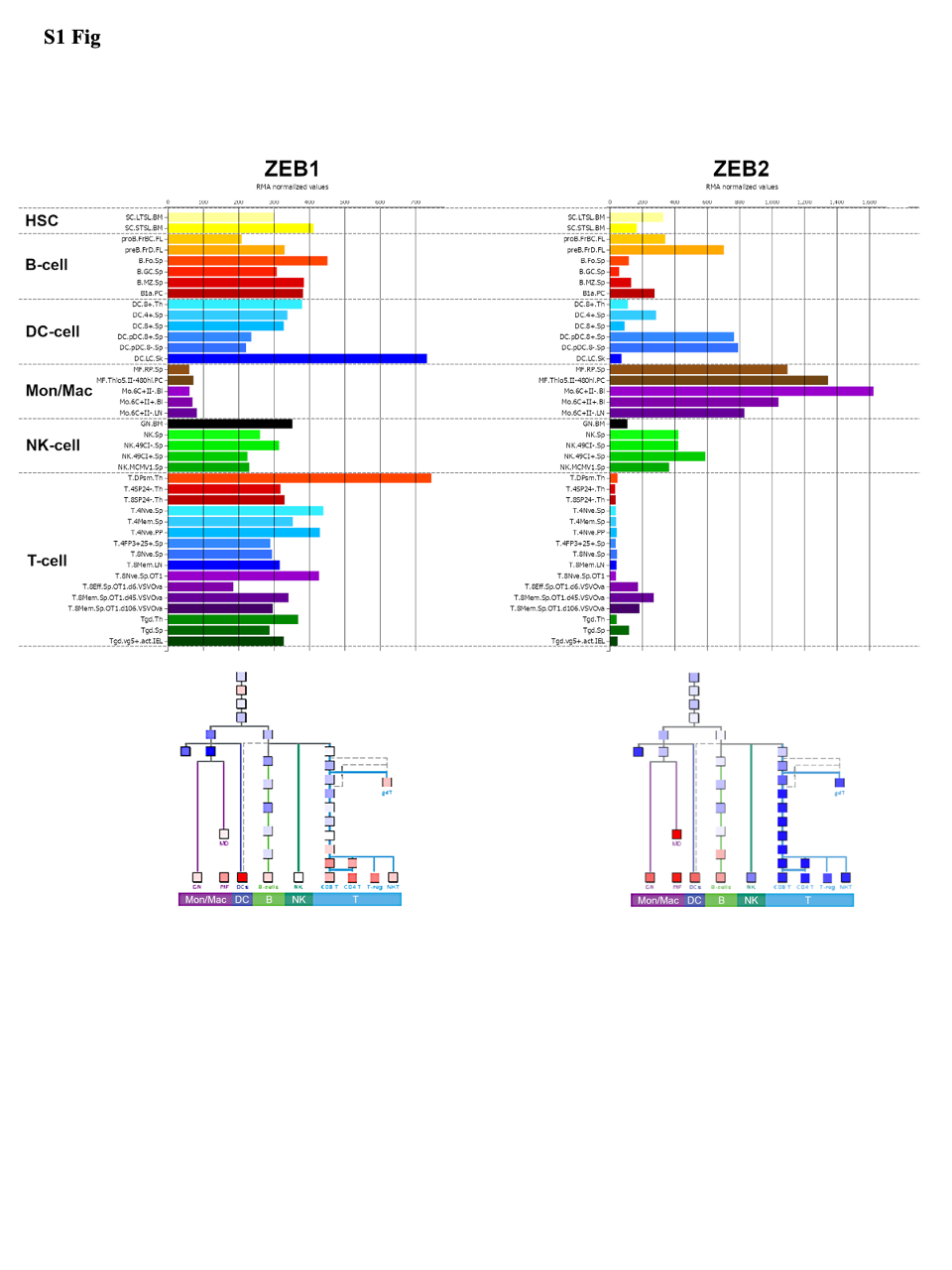

Supplement: S1 Fig — ImmGen normalized RNA expression data from adult mouse hematopoietic system for Zeb1 and Zeb2 in various hematopoietic (sub) lineages (top) and lineage hierarchy highlighting common/differential expression between Zeb1 and Zeb2 (bottom). Here, increased relative expression is highlighted as red and low expression is indicated in blue. Data generated using online tools at https://www.immgen.org/. (TIFF) [file pbio.3001394.s001.tiff]

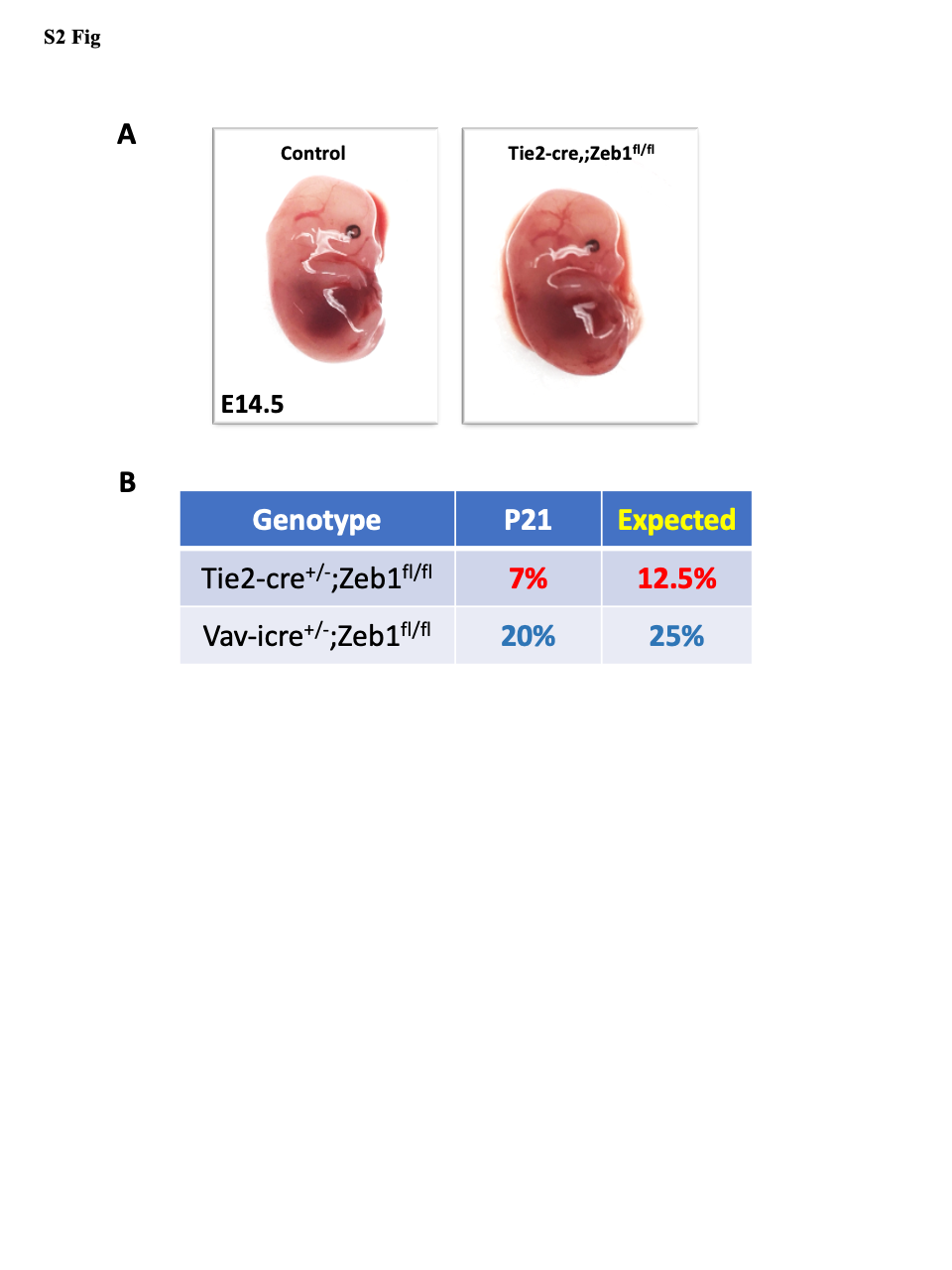

Supplement: S2 Fig — (A) Phenotypically normal control and Tie2-Cre; Zeb1fl/fl embryo at E14.5. (B) Table of expected and observed Tie2 and Vav-iCre; Zeb1fl/fl mice at P21. E14.5, embryonic day 14.5; P21, postnatal day 21. (TIFF) [file pbio.3001394.s002.tiff]

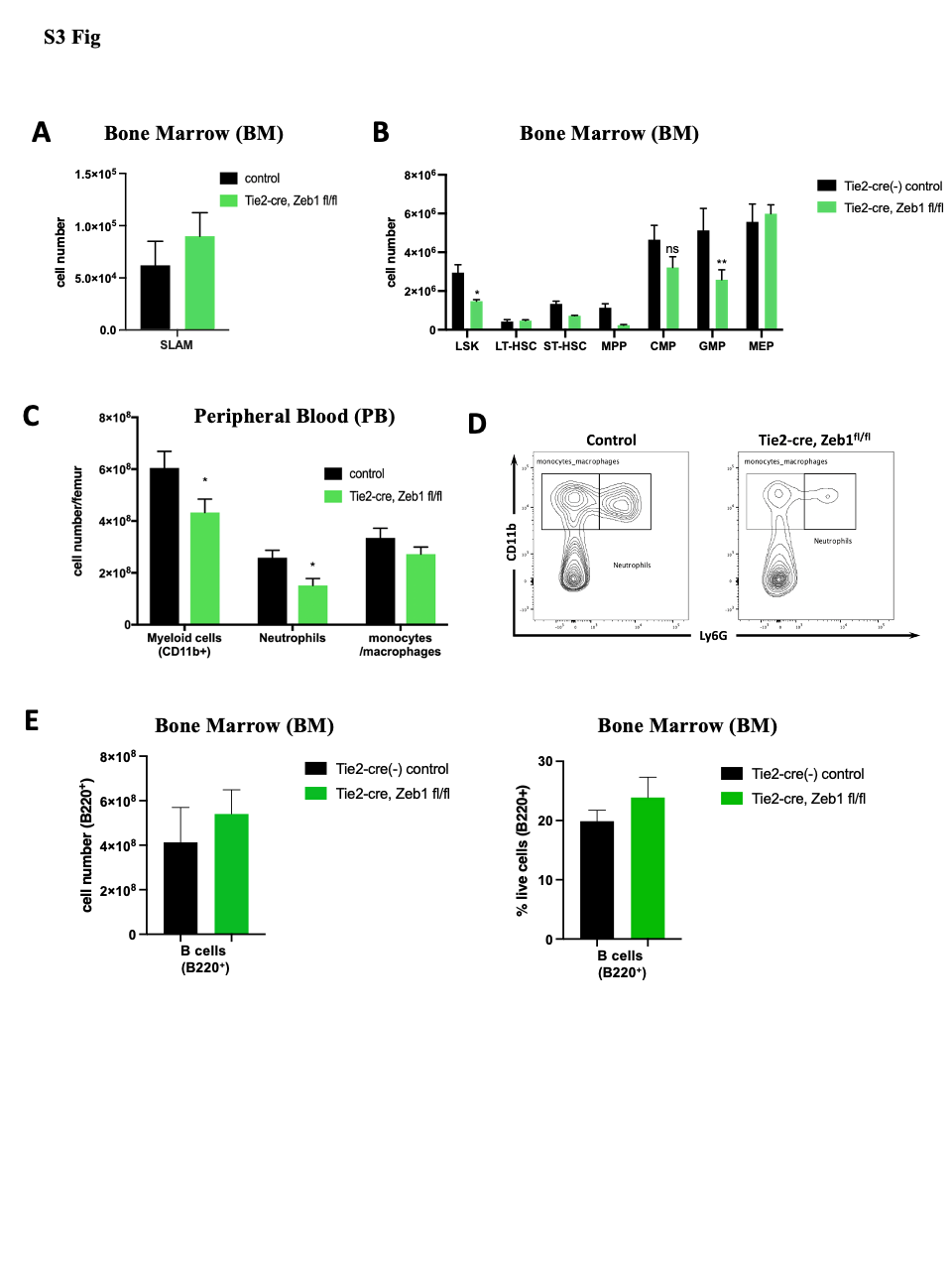

Supplement: S3 Fig — (A) SLAM marker expression showing similar numbers of LT-HSCs (CD150+CD48−) in Tie2-Cre, Zeb1 null and control Cre- reconstituted BM. (B) Flow cytometric analysis of HSPC populations within the BM of Zeb1-deficient mice identified significant decreases (*p < 0.05) in overall LSK (lin−cKit+Sca1+) numbers but no significant decrease in the total number of stem cells; LT-HSCs (lin−cKit+Sca1+CD34−Cd125−), ST-HSCs (lin−cKit+Sca1+Cd34+Cd135−), and MPPs (lin−cKit+Sca1+Cd34+Cd135+). MPP were analyzed by FcgammaR, CD34 expression to further define MEP, GMP, and CMP populations. A significant decrease in total number of GMPs but no significant changes were observed in total numbers of CMP or MEPs in Zeb1-deficient BM compared to controls. (C) Flow cytometric analysis of PB of reconstituted mice showed defects in Zeb1 null HSPC contribution to myeloid cells (Cd11b+) including monocytic (Cd11b+Ly6G−) and NEU (Cd11b+Ly6G+) lineage cells. Here, absolute cell number/femur is given. (D) Representative cytometry plot of data shown in (C). (E) B220 B cell marker analysis showing no significant differences in % or total B cells in Zeb1 null and control reconstituted BM. Here, absolute cell number/femur is given. Error bars indicate SD of the mean (n = 4 per group, *p < 0.05). Raw data behind graphs are included in A of S1 Data. BM, bone marrow; HSPC, hematopoietic stem and progenitor cell; LSK, Lin−Sca1+cKit+; LT-HSC, long-term HSC; MPP, multipotent progenitor; PB, peripheral blood; ST-HSC, short-term HSC. (TIFF) [file pbio.3001394.s003.tiff]

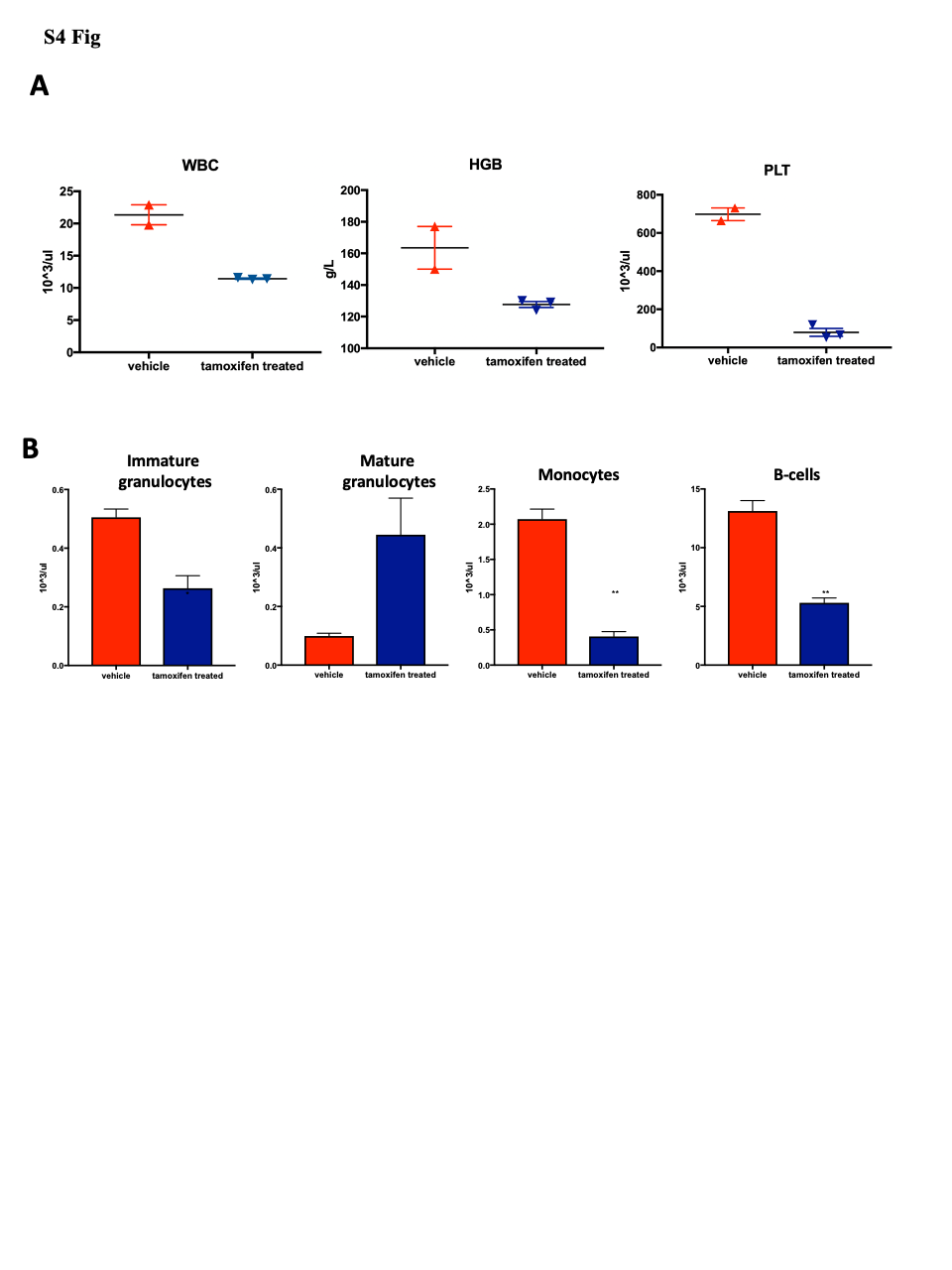

Supplement: S4 Fig — (A) HCT analysis showing decreased WBC, HGB, and PLT numbers as well as (B) decreased numbers of granulocytes, monocytes, and B cells but increases in mature granulocytes associate with tamoxifen-inducible deletion of Zeb2 in the adult BM. These phenotypes were previously observed in interferon induced Mx1-Cre mediated deletion of Zeb2 [17]. Raw data behind graphs are included in G of S1 Data. BM, bone marrow; HCT, hematocrit; HGB, hemoglobin; PLT, platelet; WBC, white blood cell. (TIFF) [file pbio.3001394.s004.tiff]

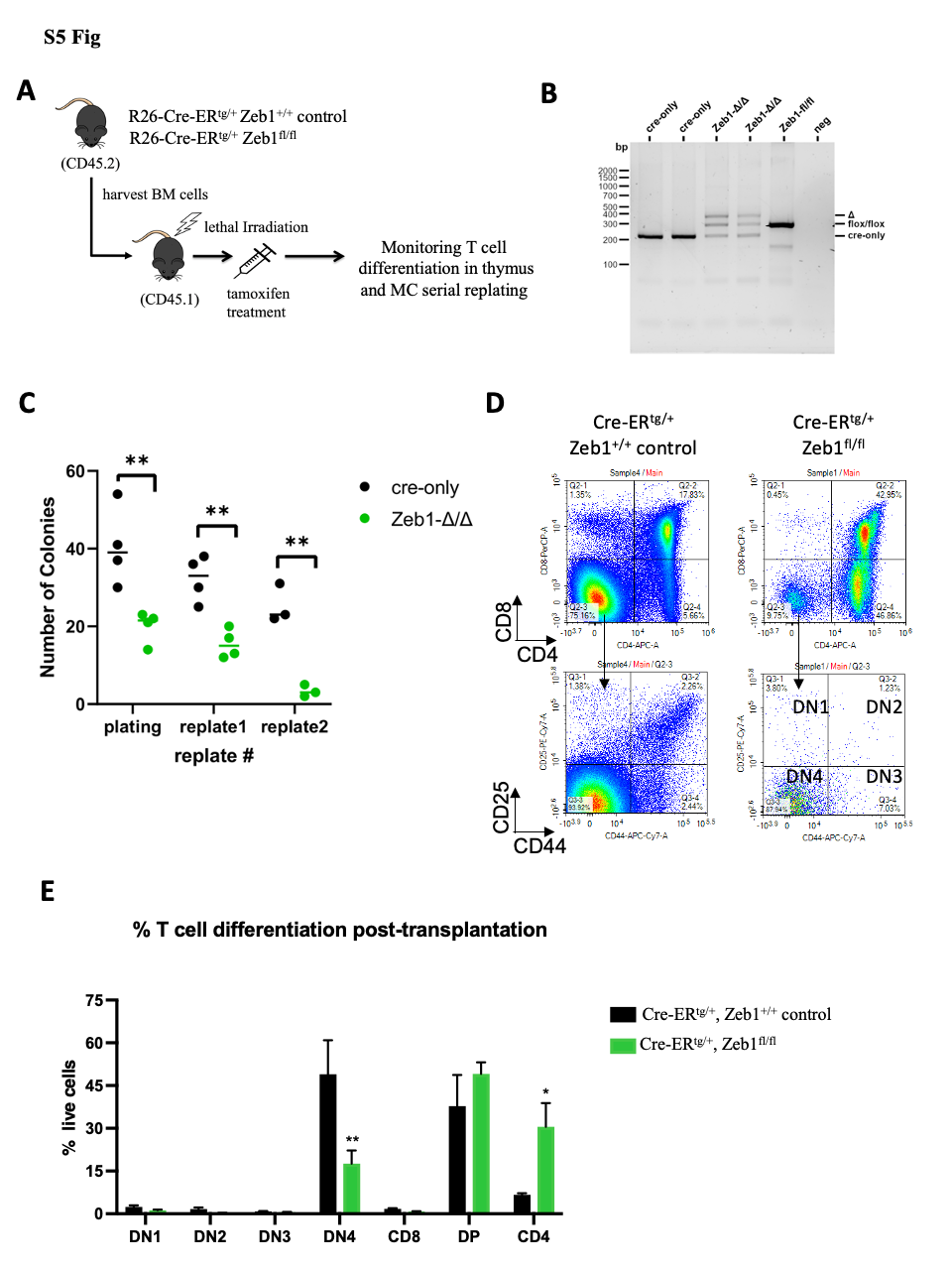

Supplement: S5 Fig — (A) Schematic of BM reconstitution experiments and analysis preformed after Cre-mediated deletion of Zeb1. (B) PCR gel analysis of genomic DNA from total BM of tamoxifen-driven excision of wt (Cre only) or Rosa26-Zeb1fl/fl mice. The upper band corresponds to deletion of Zeb1 loci (Δ, approximately 380 bp), middle band indicates the amplification of flox/flox allele (approximately 300 bp), and lower band corresponds to the amplification of the wt Zeb1 locus (Cre only, approximately 220 bp). (C) HSPCs isolated from Zeb1Δ/Δ BMs show decreased numbers of colonies in methylcellulose-based colony assays at the first plating that further decreases at the first (replate1) and secondary replating (replate2) compared to Cre negative controls. Data are represented as mean + SD from 2 biological replicates per condition, each one consisting in 2 technical replicates. *p < 0.05; **p < 0.01, nonparametric t test. (D) Representative flow cytometric analysis of thymus showing overall decreases in thymic cellularity and (E) significantly decreased DN4 (CD25−CD44−) progenitors and aberrantly expanded CD4+ T cells. N = 3/genotype *p < 0.05; **p < 0.01. Raw data behind graphs are included in H of S1 Data. BM, bone marrow; HSPC, hematopoietic stem and progenitor cell; wt, wild-type. (TIFF) [file pbio.3001394.s005.tiff]

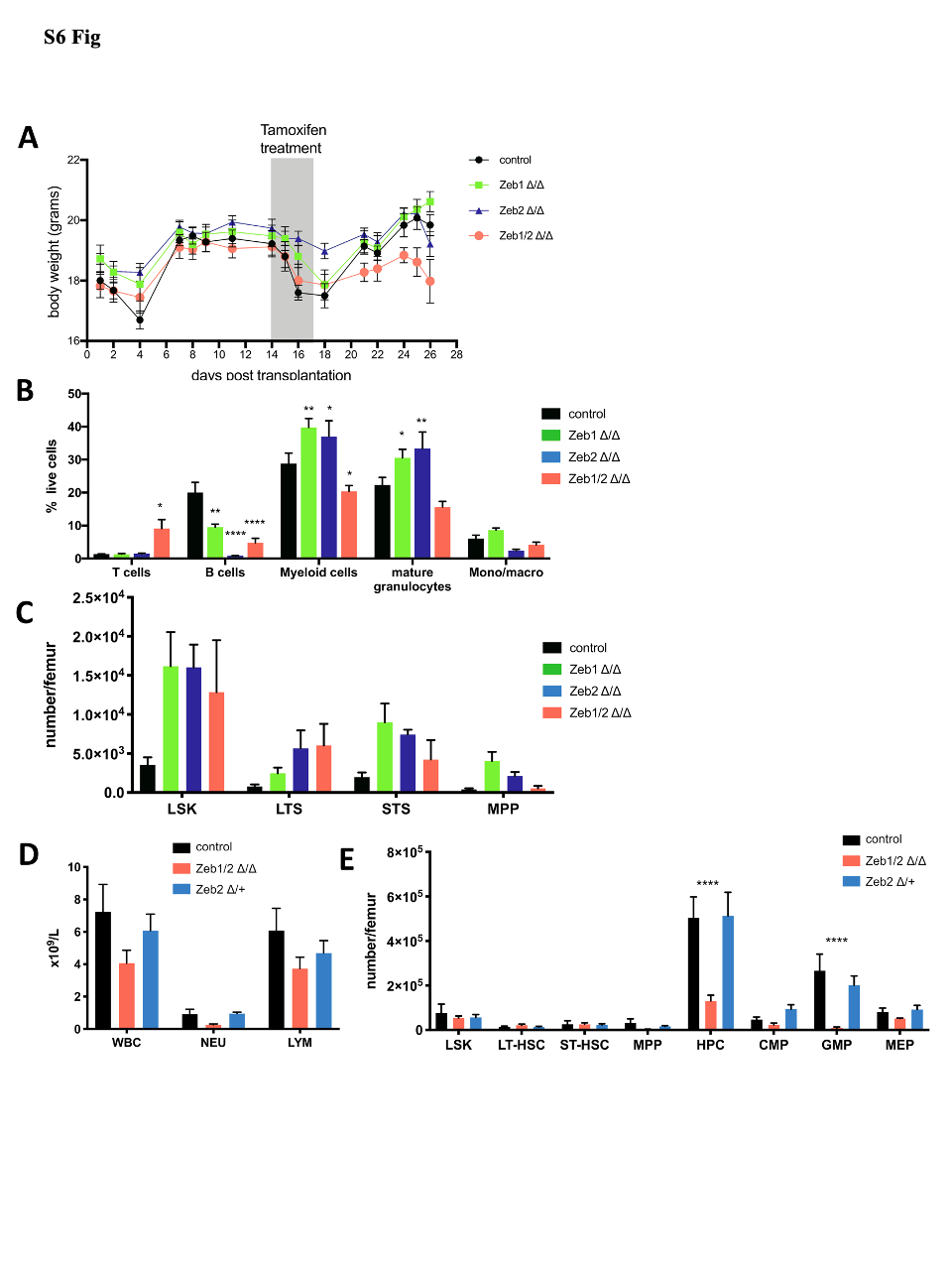

Supplement: S6 Fig — (A) Weight changes over time associated with tamoxifen mediated deletion of Zeb1, Zeb2, Zeb1/2, or Cre negative control mice. (B) Flow cytometric analysis showing fluctuating changes in hematopoietic system associated with tamoxifen mediated deletion of Zeb1, Zeb2, or both Zeb1 and Zeb2 10 days after the last dose of tamoxifen. Data presented are as a percentage of live cells. (C) Flow cytometric analysis of HSPCs in the BM 10 days after tamoxifen treatment showing alterations in total cell number/femur following deletion of Zeb1, Zeb2, or Zeb1 and Zeb2. (D) Normalization of HCT and (E) HSPC populations associated with the maintenance of a single Zeb2 allele (blue bars) compared to Zeb1/2Δ/Δ DKOs. HSPC results given are in cell number/femur. Bars in panels represent mean ± SD, n = 5 per group; *p < 0.05; **p < 0.01; ****p < 0.0001, Dunnett multiple comparisons test. Raw data behind graphs are included in I of S1 Data. BM, bone marrow; DKO, double knockout; HCT, hematocrit; HSPC, hematopoietic stem and progenitor cell; PB, peripheral blood. (TIFF) [file pbio.3001394.s006.tiff]

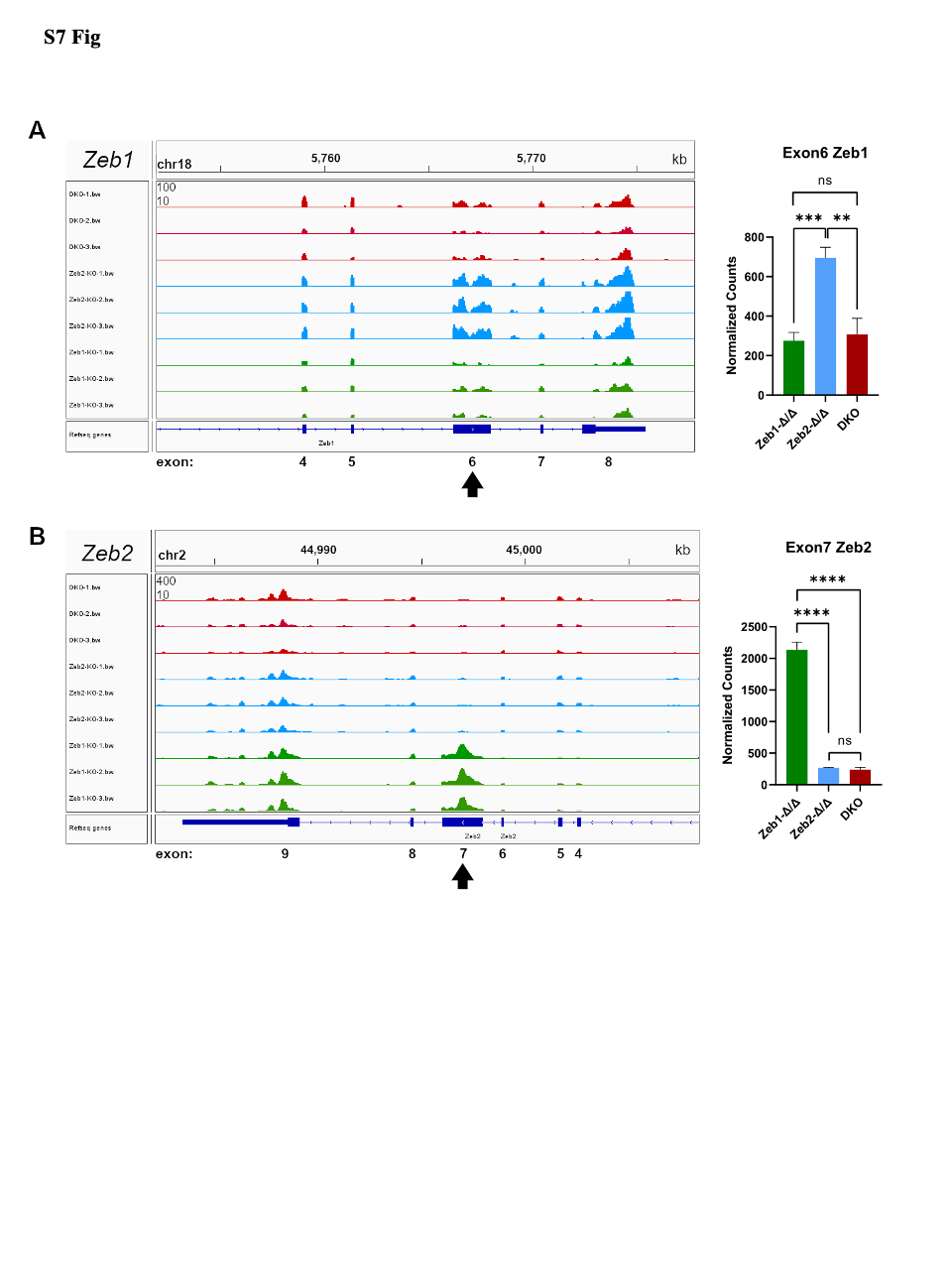

Supplement: S7 Fig — (A) (Left) IGV snapshot of 1× normalized BigWig tracks derived from alignments of Zeb1Δ/Δ, Zeb2Δ/Δ and DKO (Zeb1Δ/Δ; Zeb2Δ/Δ) LSK+ cells RNA-seq in the Zeb1 locus. From bottom to top, Zeb1Δ/Δ tracks are colored in green, Zeb2Δ/Δ tracks are colored in blue, and DKO tracks are colored in red. Genomic scale is expressed as kilobases. Exon numbers are indicated in bold letters, and the arrow indicated the tamoxifen-mediated excised floxed exon. (Right) Normalized counts per genotype of exon 6 of Zeb1. Error bars indicate SD of the mean (n = 3 per group, **p < 0.01, ***p < 0.001, ns = nonsignificant, nonparametric t test). (B) (Left) Same as (A, left) around the Zeb2 locus. Exon numbers are indicated in bold letters, and the arrow indicates the tamoxifen-mediated excised floxed exon. Genomic scale is expressed as kilobases. (Right) Same as (A, right) for the exon 7 of Zeb2. Error bars indicate SD of the mean (n = 3 per group, ****p < 0.0001, ns = nonsignificant, nonparametric t test). Raw data behind graphs are included in S6 Table. DKO, double knockout; LSK, Lin−Sca1+cKit+; RNA-seq, RNA sequencing. (TIFF) [file pbio.3001394.s007.tiff]

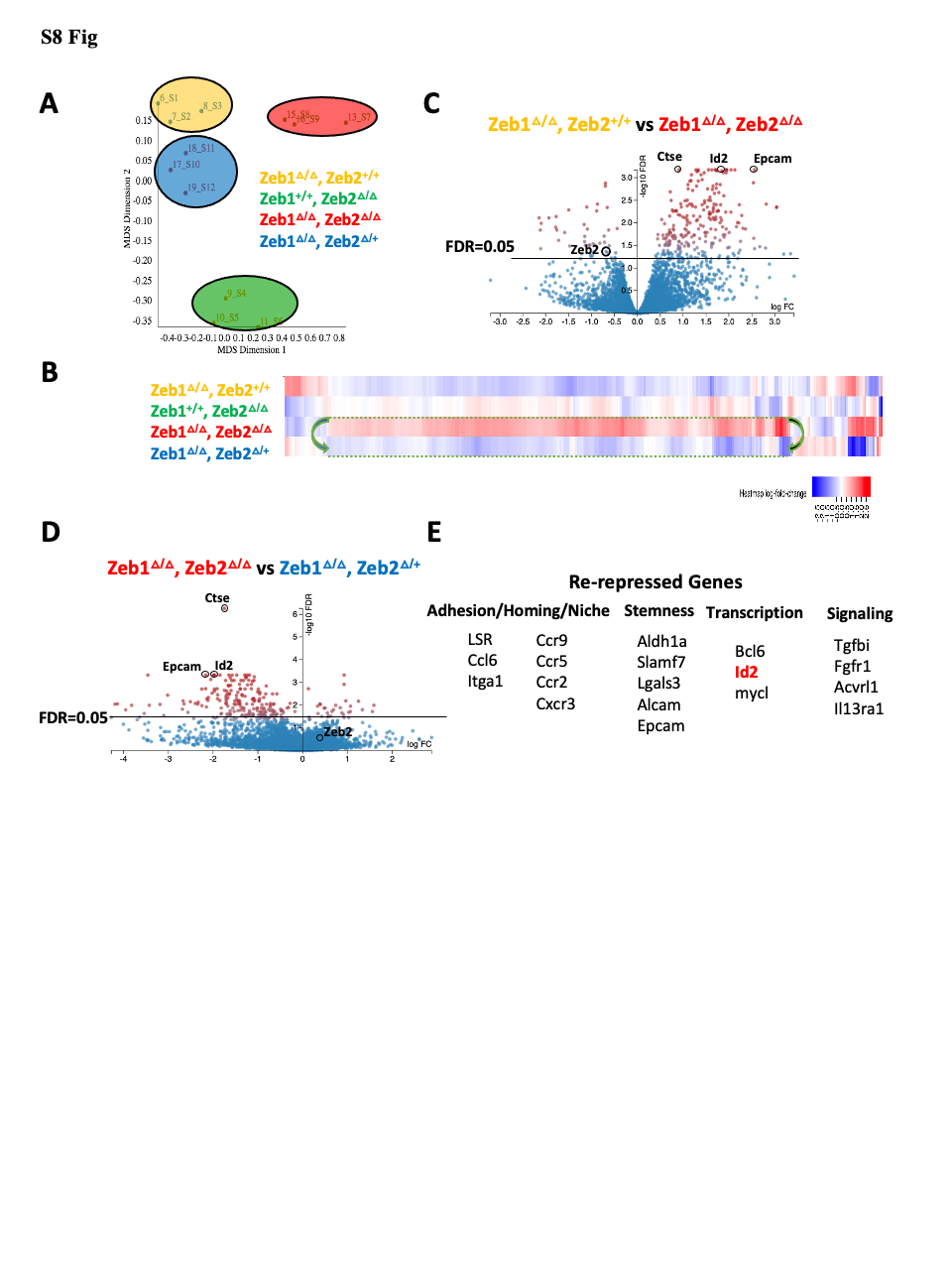

Supplement: S8 Fig — (A) PCA of DEGs showing clustering of samples based upon genotype. Zeb1Δ/Δ Zeb2+/+ samples (yellow) cluster more closely together with Zeb1Δ/Δ Zeb2Δ/+ samples (blue). Zeb1Δ/Δ Zeb2Δ/Δ DKO (red) and Zeb1+/+Zeb2Δ/Δ (green) samples cluster father apart because they are more genetically diverse. (B) Gene expression heatmap showing that Zeb1/2 DKO samples have many genes that become up-regulated compared to either Zeb1 or Zeb2 single deletion alone and these genes appear to be normalized if even a single Zeb2 allele is present in Zeb1Δ/ΔZeb2Δ/+ samples. (C) Volcano plot of DEGs highlighting differences in gene expression between Zeb1Δ/ΔZeb2+/+ and Zeb1Δ/ΔZeb2Δ/Δ DKO samples. Highlighted are 3 genes known to be repressed by Zeb2 including Ctse, Id2, and Epcam. Moreover, Zeb2 is highlighted as being down-regulated between the 2 samples. (D) Volcano plot showing how maintained presence of single Zeb2 allele in Zeb1Δ/ΔZeb2Δ/+ samples can lead to repression or normalization of up-regulated genes observed in Zeb1/2 DKO samples. FDR rates for (C) and (D) was 0.05. (E) Highlighted Zeb2 DEGs and rough grouping of biological processes associated with each gene. Raw data behind plots are included in S2 Table. DEG, differentially expressed gene; DKO, double knockout; FDR, false discovery rate; LSK, Lin−Sca1+cKit+; PCA, principle component analysis. (TIFF) [file pbio.3001394.s008.tiff]

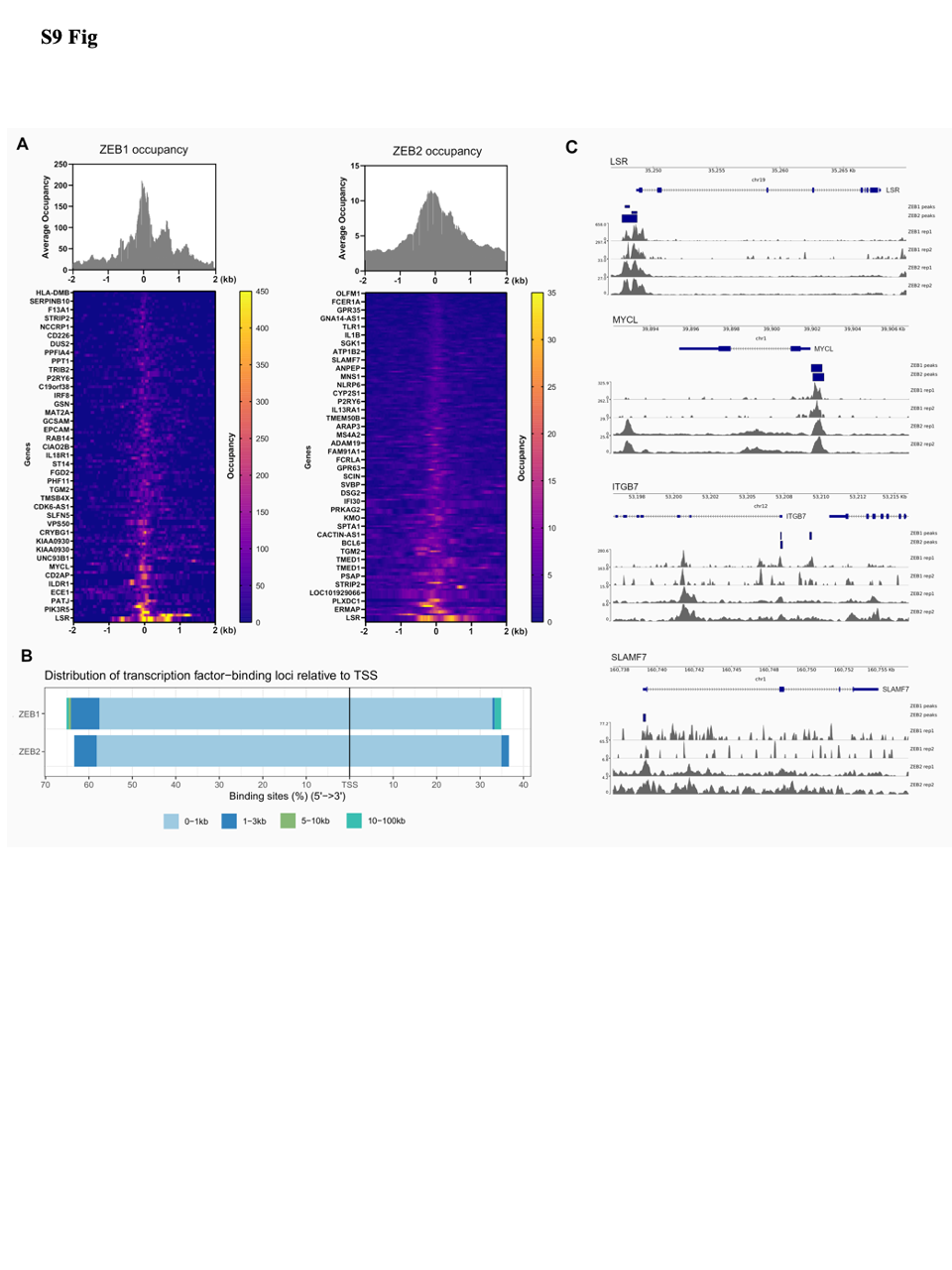

Supplement: S9 Fig — (A) Plot of the average occupancy and the occupancy profiling of ZEB1 and ZEB2 in GM12878 and K562 cell lines, respectively, in 2 different ChIP-seq experiments. Each row represents a human homolog matching mouse DEGs found in LSK cells. The plots are centered at the first ATG of every gene, and rows are sorted by occupancy values from the smallest (top) to highest (bottom) in a 4-kb window. ChIP-seq data were aligned against hg38 human genome, and BigWig files were obtained from each BAM file by using deeptools bamCoverage tool. Each BigWig file was normalized to 1× method using the mappable human genome size. The colormap used in the heatmaps was jet and the missing data color in the plots was dark blue. (B) Distribution of transcription factor binding loci relative to the TSS of the plotted genes in (A) upstream and downstream from the TSS of genes, expressed as percentages, obtained with the ChIPseeker R package. Distance to the TSS was plotted with distinctive colors. (C) Human gene models and BigWig tracks from the top-enriched gene LSR including MYCL, ITGB7, and SLAMF7 genes presenting ZEB1 and/or ZEB2 peaks in all ChIP-seq datasets (including technical replicates). Raw data behind plots are included in J of S4 Table. ChIP-seq, Chromatin Immunoprecipitation Sequencing; DEG, differentially expressed gene; LSK, Lin−Sca1+cKit+; TSS, transcriptionally start site. (TIFF) [file pbio.3001394.s009.tiff]

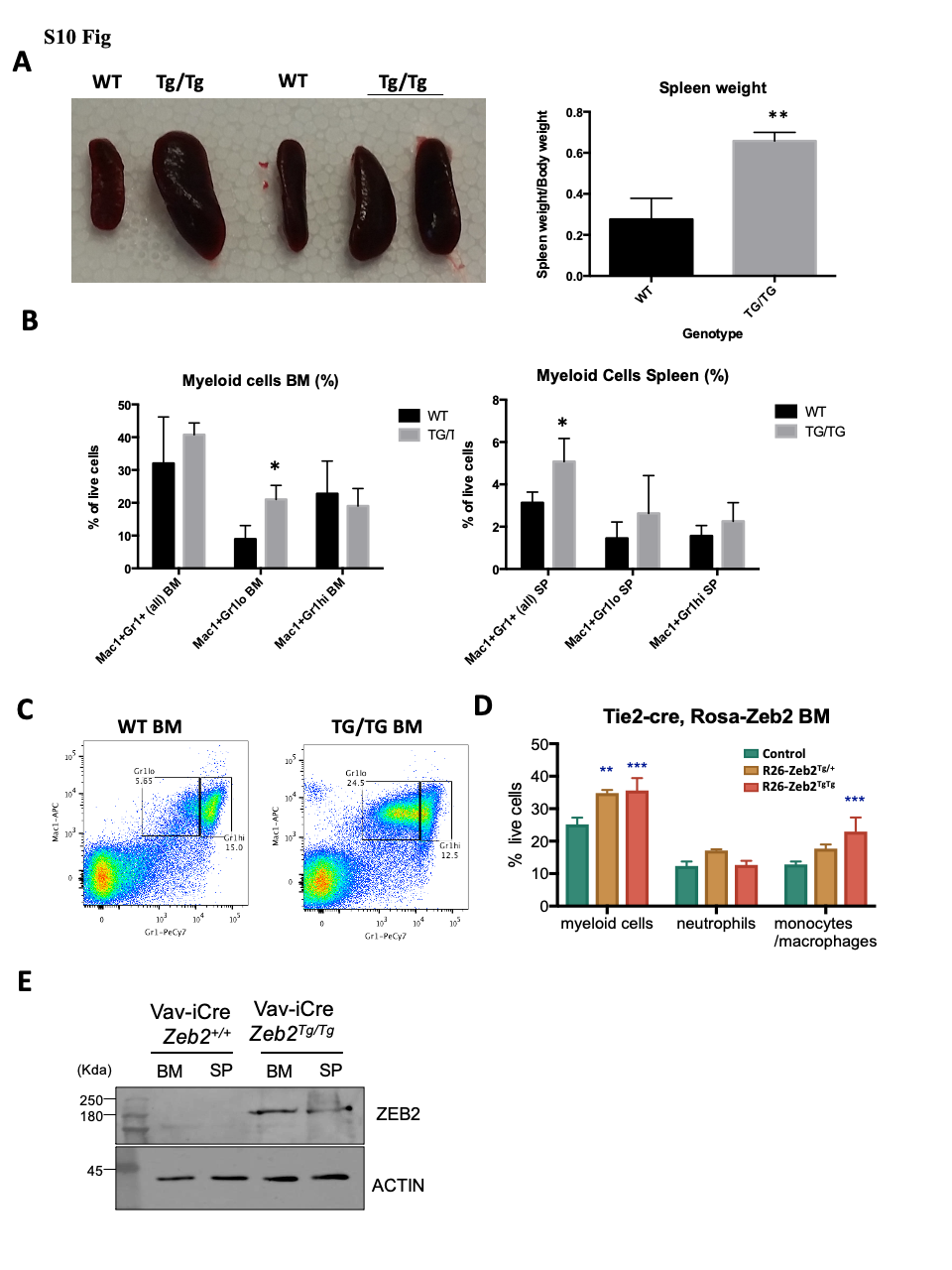

Supplement: S10 Fig — (A) Increased spleen size/extramedullary hematopoiesis seen in Tie2-Cre; Zeb2Tg/Tg transgenic mice (left panel) showing roughly doubling in size compared to body weight (right panel). (B) Flow cytometric analysis showing increased myeloid cells (CD11b+, Gr1lo) in the BM (left) and spleen (right). (C) Representative flow cytometry plot showing increased CD11b+, Gr1lo myeloid cells in the BM of Tie2-Cre; Zeb2Tg/Tg mice. (D) Summary graph of increased myeloid cells (CD11b+, Gr1lo) in heterozygous Tie2-Cre; R26-Zeb2Tg/+ and homozygous R26-Zeb2Tg/Tg BM cells as well as increased monocytes (CD11b+, Lys6G-) present in R26-ZebTg/Tg BM cells. (E) Western blot analysis showing increased ZEB2 protein expression in Vav-iCre; Zeb2Tg/Tg BM and spleen compared to Cre-only controls. Data are represented as mean + SD from 3 biological replicates/genotype except in (A) where only 2 wt controls were used. Male and female mice were analyzed ranging in age from 4 to 10 months of age. Raw data behind graphs and western blot are included in J of S1 and S2 Data, respectively. *p < 0.05; **p < 0.01, ***p < 0.001, nonparametric t test. BM, bone marrow; wt, wild-type. (TIFF) [file pbio.3001394.s010.tiff]

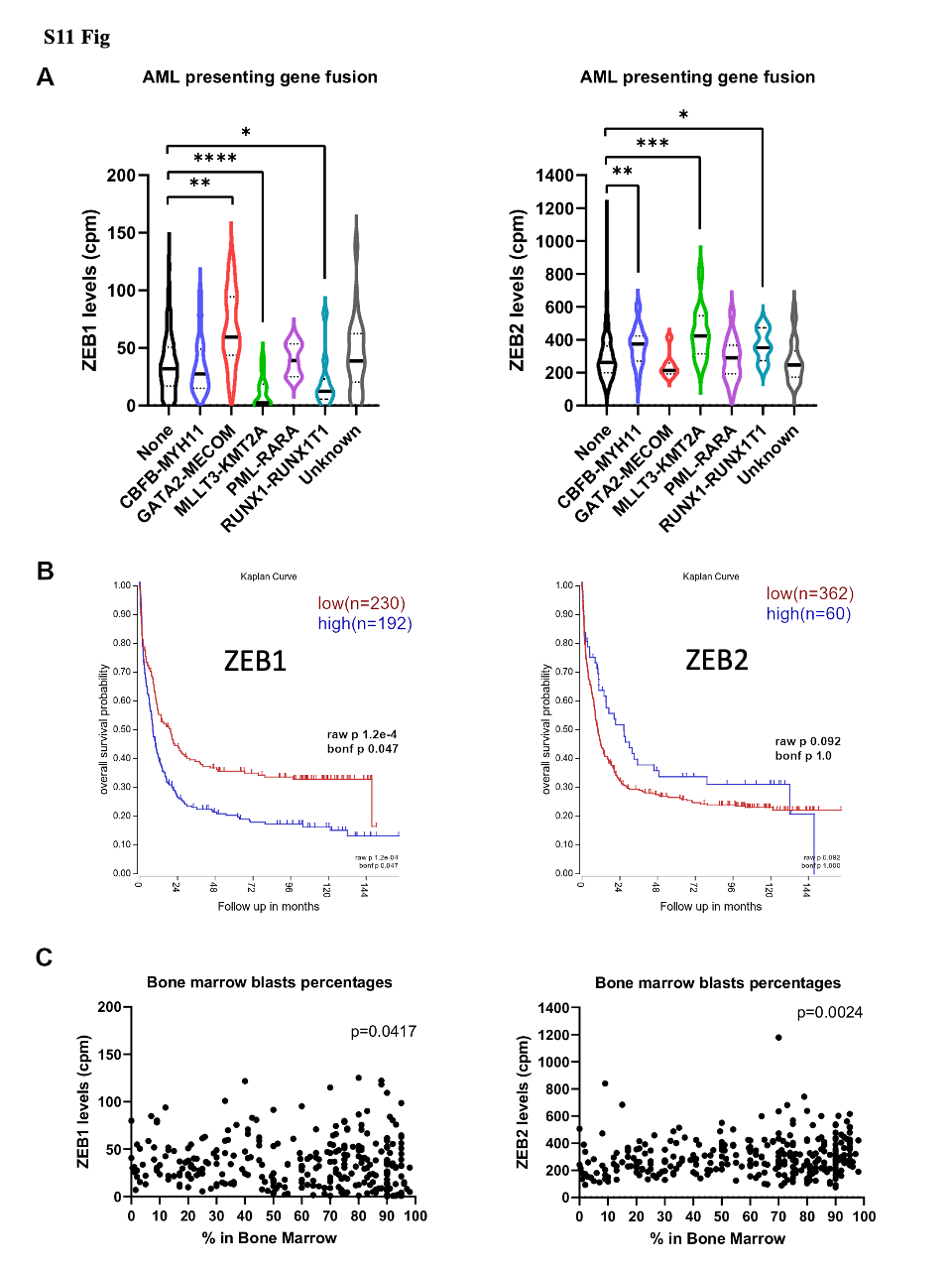

Supplement: S11 Fig — (A) Violin plots depicting normalized gene counts as TPM of ZEB1 (left) and ZEB2 (right) gene expression across AML samples aggregated by the presence of genomic gene fusions present in human AML [23]. Median including 75% and 25% quartiles are denoted from top to bottom as dashed lines. (B) Kaplan–Meier analysis of samples containing high (blue line) or low (red line) expression of ZEB1 (left) and ZEB2 (right). The appropriate cutoff was defined through a scanning method implemented in the R2 database (http://r2.amc.nl). Bonferroni corrected p-values determine that higher ZEB1 statistically reduce the overall survival probability (S9B Fig, left), while higher expression of ZEB2 did not cause this effect. *p < 0.05; **p < 0.01, ***p < 0.001, Bonferroni-corrected nonparametric t test. (C) Increased expression of both ZEB1 (left) and ZEB2 (Right) appear to significantly correlate with increased numbers of leukemic blasts present in AML populations (p = 0.0417 and p = 0.0024, respectively). Raw data behind plots are included in S7 Table. AML, acute myeloid leukemia; TPM, transcripts per million. (TIFF) [file pbio.3001394.s011.tiff]

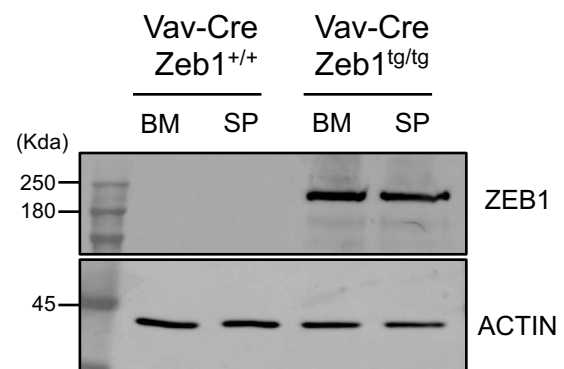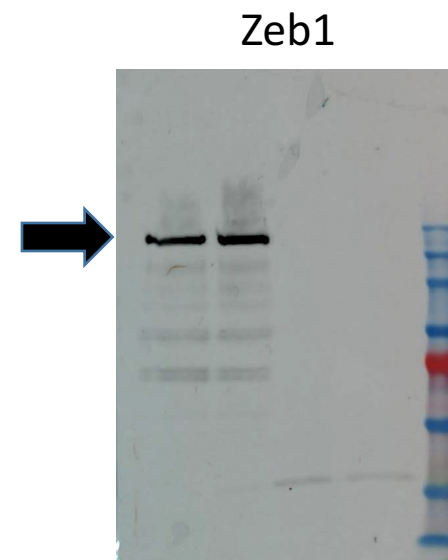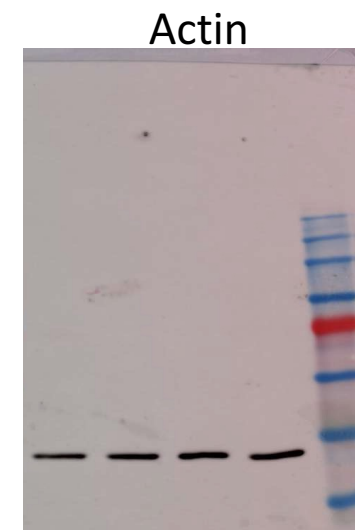

**Fig 8 D**

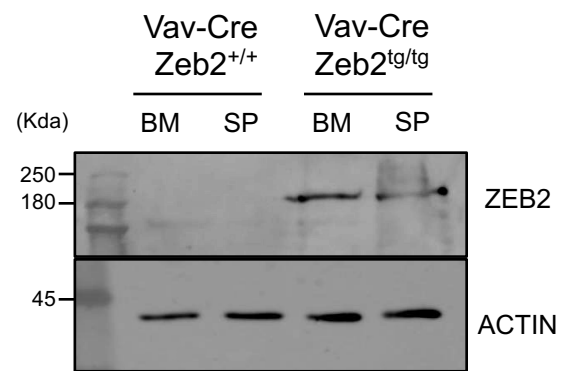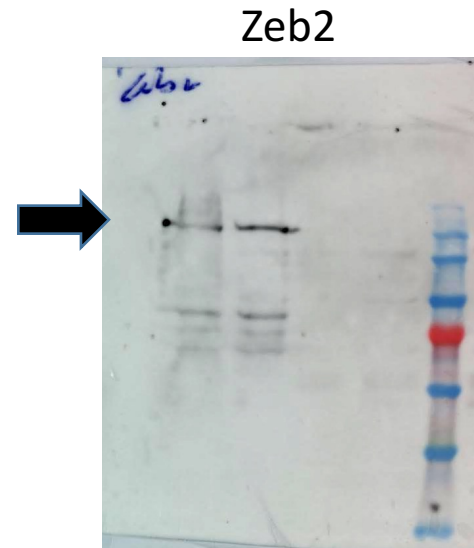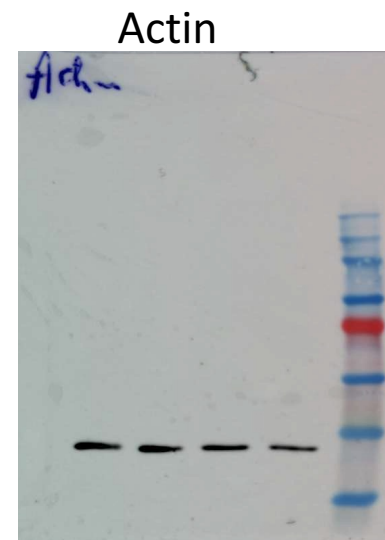

Supplement: S2 Data — (PDF) [file pbio.3001394.s021.pdf]
