# Supplementary material for: Interplay between the EMT transcription factors ZEB1 and ZEB2 regulates hematopoietic stem and progenitor cell differentiation and hematopoietic lineage fidelity
Source: PLoS Biol. 2021 Sep 22;19(9):e3001394. doi: 10.1371/journal.pbio.3001394 (PMC8489726; doi:10.1371/journal.pbio.3001394)
Supplement: S1 Text — (DOCX) [file pbio.3001394.s012.docx]

Interplay between the EMT transcription factors ZEB1 and ZEB2 regulates hematopoietic stem and progenitor cell differentiation and hematopoietic lineage fidelity.

Jueqiong Wang^1^, Carlos Farkas^2,3^, Aissa Benyoucef^2,3^, Catherine Carmichael^1^, Katharina Haigh^2,3^, Nick Wong^1^, Danny Huylebroeck^4,5^, Marc P. Stemmler^6^, Simone Brabletz^6^, Thomas Brabletz^6^, Christian Nefzger^7,8,9^, Steven Goossens^10,11,12^, Geert Berx^10,11^, Jose M. Polo^6,7,8^, Jody J. Haigh^1,2,3*^

1. Australian Centre for Blood Diseases, Monash University, Melbourne, Australia

2. Department of Pharmacology and Therapeutics, Rady Faulty of Health Sciences, University of Manitoba, Winnipeg, Manitoba, Canada

3. CancerCare Manitoba Research Institute, Winnipeg, Manitoba, Canada

4. Department of Cell Biology, Erasmus University Medical Center, Rotterdam, The Netherlands.

5. Department of Development and Regeneration, KU Leuven, Leuven, Belgium.

6. Department of Experimental Medicine 1, Nikolaus-Fiebiger-Centre for Molecular Medicine, FAU University Erlangen-Nürnberg, Erlangen, Germany

7. Department of Anatomy and Developmental Biology, Monash University, Melbourne, Australia

8. Development and Stem Cells Program, Monash Biomedicine Discovery Institute, Melbourne, Australia

9. Australian Regenerative Medicine Institute, Monash University, Melbourne, Australia

10. Molecular and Cellular Oncology Laboratory, Department of Biomedical Molecular Biology, Ghent University, Ghent, Belgium.

11. Cancer Research Institute Ghent (CRIG), Ghent University, Ghent, Belgium

12. Department of Diagnostic Sciences, Ghent University and University Hospital, Ghent, Belgium.

*Corresponding Author:

Professor Jody Jonathan Haigh

Department of Pharmacology and Therapeutics

Rady Faculty of Health Sciences

University of Manitoba

Senior Scientist

CancerCare Manitoba Research Institute, ON5029

675 McDermot Ave

Winnipeg, MB

Canada, R3E 0V9

Tel: 204-787-2134

Mob: 204-995-9042

[E-mail: jody.haigh@umanitoba.ca](mailto:jody.haigh@umanitoba.ca)

**Supplementary Methods and Materials**

**Bulk RNA-seq analysis**

Initial RNAseq data was processed and analyzed using Degust software tools developed at Monash Univesity (https://degust.erc.monash.edu/). Subsequently, we aligned single-end reads using HISAT2 aligner version 2.0.4, using the -U option [1]. We employed GENCODE mouse genome build GRCm38.p6 as mouse reference genome in FASTA format. We sorted and indexed resulting BAM files with Samtools program [2]. We quantified aligned reads in genes using featureCounts program [3], employing gene transfer format file gencode.vM24.chr_patch_hapl_scaff.annotation.gtf from GENCODE GRCm38.p6 build, obtaining a count table of 56246 genes for differential expression analysis. We assessed differential expression analysis using the unconditional exact test from edgeRun R package with 50000 iterations, setting FDR<0.1 as differentially expressed gene (DEG) cutoff [4]. We converted edgeRun normalized gene counts from each replicate to Z-score by directly running pheatmap R package on the transformed expression levels (<https://cran.r-project.org/web/packages/pheatmap/index.html>). We obtained heatmap plots of Z-score values across replicates by using GraphPad Prism 8 program (<https://www.graphpad.com/scientific-software/prism/>). We obtained GO terms and Network analysis by inputting list of DEGs in the STRING web-server for functional enrichment analysis version 11 [5]. GO terms were obtained from a combined source from BioCyc[6], GO [7], KEGG [8], and Reactome [9]. Networks were constructed using the highest confidence (0.900) combined score, computed by combining the probabilities from the different evidence channels and corrected for the probability of randomly observing an interaction. We clustered networks using a MCL inflation parameter of 3.

**Single cell RNA-seq analysis**

We downloaded Nestorowa et al [10] sequencing experiment (BioProject: PRJNA322317) and quantified gene expression across sequenced cells using the *salmon quant* command from Salmon [11] setting the following parameters: --numBootstraps 30, -l SR and -p 40. We used the same mouse reference genome and annotation as described in the bulk RNA-seq analysis. We merged gene quantification using the *salmon quantmerge* command with the following parameters: --quants * --genes --missing arg 0 -o salmon_counts.tab. We replaced ENSEMBL gene id with Gene names in the salmon_counts.tab output file with a custom BASH script. Cell types (LT-HSC, HSPC and Prog) and correspondent cell fates sublineages were downloaded from <http://blood.stemcells.cam.ac.uk/data/all_cell_types.txt>. The latter matrix was converted in a catergorical binary matrix and inputted along with the count table (as matrix) in R. We used Monocle3 package to construct a single cell dataset object and further analyze the data [12, 13]. To discover clusters, we calculated the Principal component analysis (PCA) of the data using the Uniform Manifold Approximation and Projection (UMAP) reduction method [14]. Then, we identified all cells close to the starting point and computed trajectories with standard Monocle3 commands. We extracted pseudotimes and cell weights for the tradeSeq program [15] and we fit gene expressing against as negative binomial generalized additive model (NB-GAM) using a K=3 as number of knots. We estimated the number of knots using the *evaluateK* tradeSeq function, based on a diagnostic plot using the Akaike Informaction Criterion (AIC). Then, we constructed a slingshot object [16] and fit gene expression using the *fitGAM* tradeSeq function. We plotted smoother curves for Zeb1 and Zeb2 in reduced dimensions along pseudotime with *plotGeneCount* command for tradeSeq.

**Zeb1 and Zeb2 Exome excision quantification of RNA-seq data**

To produce IGV snapshots of normalized coverage per sequenced sample, we obtained BigWig files from the correspondent aligned bam files by using the deeptools bam Coverage tool [17]. Each bigwig file was normalized to 1x method using an effective genome size of 2652783500 base pairs and a bin length of 50 bp.

To obtain all exons from the mouse genome, we downloaded the mouse GENCODE GTF annotation file release M25 (gencode.vM25.chr_patch_hapl_scaff.annotation) from the GENCODE project [18] We quantified all exon reads by using featureCounts program [3], using the following options: -t exon -g exon_id -O. A count table was parsed from featureCounts using standard UNIX commands, that was loaded in the R environment. After calculating normalizing factors and estimated dispersions with edgeR package [19], we obtained a normalized count table as follows:

norm_counts.table <- t(t(d$pseudo.counts)*(d$samples$norm.factors))

write.table(norm_counts.table, file="./Zeb1_vs_Zeb2_vs_DKO_normalizedCounts.tab", sep="\t", quote=F)

We fetched exon seven of *Zeb2* gene (ENSMUSE00000275656) and exon six of Zeb1 gene (ENSMUSE00001041497) on the normalized data (Zeb1_vs_Zeb2_vs_DKO_normalizedCounts.tab) and we plotted the normalized counts corresponding to these exons as a barplot, using three replicates per Zeb1^Δ/Δ^, Zeb2^Δ/Δ^ and Zeb1^Δ/Δ^ ; Zeb2^Δ/Δ^ DKO genotypes, respectively. To perform statistical assumptions, we applied non-parametric t-test (n=3 per group, *p<0.05, **p<0.01, *** p<0.001 and ****p<0.0001).

**ChIP-seq analysis**

We aligned Illumina ChIP-seq reads of ZEB1 in GM12878 (accessions SRR351657 and SRR351658, respectively) and ZEB2 in K562 (accessions SRR5111418 and SRR5111419, respectively) against to the human genome (hg38 assembly) by using bowtie2 aligner with default settings [20]. We sorted and indexed correspondent BAM files with SAMtools [2]. We called peaks from the resulting BAM files using MACS2 program, using an effective genome size of 2,913,022,398 base pairs, read extension size of 151, and peak detection based on False Discovery Rate (q-value) less than 0.05. We obtained bigwig files from aligned BAM files using the deeptools bam Coverage tool [17]. By using deepTools computeMatrix tool, we obtained a matrix computing the coverage of each dataset according to the sort of the ChIP-seq peaks using BED files of 2-kb window, centred to the middle of the peak in each case. We plotted heatmaps from the resulting computation with the deepTools plot Heatmap tool. We also annotated ChIP-seq peaks in BED format with the ChIPseeker R Bioconductor package [21]. ChIP-seq tracks in BigWig format including associated BED files containing peaks were visualized with the IGV software [22].

**Supplementary References**

1. Kim, D., et al., *Graph-based genome alignment and genotyping with HISAT2 and HISAT-genotype.* Nat Biotechnol, 2019. **37**(8): p. 907-915.

2. Li, H., et al., *The Sequence Alignment/Map format and SAMtools.* Bioinformatics, 2009. **25**(16): p. 2078-9.

3. Liao, Y., G.K. Smyth, and W. Shi, *featureCounts: an efficient general purpose program for assigning sequence reads to genomic features.* Bioinformatics, 2014. **30**(7): p. 923-30.

4. Dimont, E., et al., *edgeRun: an R package for sensitive, functionally relevant differential expression discovery using an unconditional exact test.* Bioinformatics, 2015. **31**(15): p. 2589-90.

5. Szklarczyk, D., et al., *STRING v11: protein-protein association networks with increased coverage, supporting functional discovery in genome-wide experimental datasets.* Nucleic Acids Res, 2019. **47**(D1): p. D607-D613.

6. Paley, S. and P.D. Karp, *Update notifications for the BioCyc collection of databases.* Database (Oxford), 2017. **2017**.

7. Mi, H., et al., *PANTHER version 14: more genomes, a new PANTHER GO-slim and improvements in enrichment analysis tools.* Nucleic Acids Res, 2019. **47**(D1): p. D419-D426.

8. Kanehisa, M., et al., *KEGG: integrating viruses and cellular organisms.* Nucleic Acids Res, 2020.

9. Fabregat, A., et al., *The Reactome Pathway Knowledgebase.* Nucleic Acids Res, 2018. **46**(D1): p. D649-D655.

10. Nestorowa, S., et al., *A single-cell resolution map of mouse hematopoietic stem and progenitor cell differentiation.* Blood, 2016. **128**(8): p. e20-31.

11. Patro, R., et al., *Salmon provides fast and bias-aware quantification of transcript expression.* Nat Methods, 2017. **14**(4): p. 417-419.

12. Qiu, X., et al., *Single-cell mRNA quantification and differential analysis with Census.* Nat Methods, 2017. **14**(3): p. 309-315.

13. Trapnell, C., et al., *The dynamics and regulators of cell fate decisions are revealed by pseudotemporal ordering of single cells.* Nat Biotechnol, 2014. **32**(4): p. 381-386.

14. McInnes, L., J. Healy, and J. Melville *UMAP: Uniform Manifold Approximation and Projection for Dimension Reduction*. 2018. arXiv:1802.03426.

15. Van den Berge, K., et al., *Trajectory-based differential expression analysis for single-cell sequencing data.* Nat Commun, 2020. **11**(1): p. 1201.

16. Street, K., et al., *Slingshot: cell lineage and pseudotime inference for single-cell transcriptomics.* BMC Genomics, 2018. **19**(1): p. 477.

17. Ramirez, F., et al., *deepTools: a flexible platform for exploring deep-sequencing data.* Nucleic Acids Res, 2014. **42**(Web Server issue): p. W187-91.

18. Harrow, J., et al., *GENCODE: the reference human genome annotation for The ENCODE Project.* Genome Res, 2012. **22**(9): p. 1760-74.

19. Robinson, M.D., D.J. McCarthy, and G.K. Smyth, *edgeR: a Bioconductor package for differential expression analysis of digital gene expression data.* Bioinformatics, 2010. **26**(1): p. 139-40.

20. Langmead, B., et al., *Ultrafast and memory-efficient alignment of short DNA sequences to the human genome.* Genome Biol, 2009. **10**(3): p. R25.

21. Yu, G., L.G. Wang, and Q.Y. He, *ChIPseeker: an R/Bioconductor package for ChIP peak annotation, comparison and visualization.* Bioinformatics, 2015. **31**(14): p. 2382-3.

22. Thorvaldsdottir, H., J.T. Robinson, and J.P. Mesirov, *Integrative Genomics Viewer (IGV): high-performance genomics data visualization and exploration.* Brief Bioinform, 2013. **14**(2): p. 178-92.

23. Tyner, J.W., et al., *Functional genomic landscape of acute myeloid leukaemia.* Nature, 2018. **562**(7728): p. 526-531.
