# Supplementary material for: Interplay between the EMT transcription factors ZEB1 and ZEB2 regulates hematopoietic stem and progenitor cell differentiation and hematopoietic lineage fidelity
Source: PLoS Biol. 2021 Sep 22;19(9):e3001394. doi: 10.1371/journal.pbio.3001394 (PMC8489726; doi:10.1371/journal.pbio.3001394)
Supplement: Attachment — Submitted filename: Wang et al-Response to Reviewers.docx [file pbio.3001394.s022.docx]

We would like to start out by thanking the reviewers for their positive comments and suggestions. Wherever possible we have tried to address their comments/concerns either experimentally or through further bioinformatics analysis. Changes in the manuscript are highlighted in red in the manuscript and our response to the reviewer’s comments are highlighted in blue below.

**Reviewers’ comments**

**Rev. 1:**

Summary:
*The authors are interested in the role of transcription factors ZEB1 and ZEB2 in hematopoietic stem cell (HSC) maintenance and differentiation. The authors use a number of complex model systems to look at knockout of Zeb1 and Zeb2, along with overexpression of the genes. It is taken under consideration the time and work to perform the complex breeding. The studies define the role of Zeb1 and Zeb2 in hematopoietic stem and progenitor populations along with lineage differentiation. In addition, the authors explore the role of the genes in acute myeloid leukemia (AML) progression. Overall, the manuscript contains substantial amount of data and findings on Zeb1, and Zeb2, however important details are missing from the manuscript that would strengthen the conclusions. Unfortunately, lack of detail in portions of the manuscript, poor figure quality, and conclusions not supported by the data greatly reduces the enthusiasm. I would recommend major revisions of the manuscript.*

*Weaknesses:
* In the introduction the authors mention that ZEB1 and ZEB2 potentially contribute to development and/or maintenance of leukemia. A number of large data sets are available that look at expression and survival in AML. It would strengthen the manuscript to include expression data, especially in MLL subset of patients. Are ZEB1 and ZEB2 highly expressed in AML and is expression correlated with poor survival?*

We have attempted to address these issues within the context of our MLL-AF9 leukemic models in Figure 9 in our New Supplementary Figure 10. Based upon the large and comprehensive AML dataset published by Tyner et al., *Nature* 2018 we have examined the expression of both *ZEB1* and *ZEB2* in AML populations with 5 different driver mutations in addition to AML with unknown etiology compared with non-transformed myeloid populations. Here we found that *ZEB1* is significantly upregulated in GATA2-MECON driven AML but significantly decreased in MMLT3-KMT2A (equivalent to MLL-AF9) and RUNX1-RUNX1T1 AML. In contrast, *ZEB2* levels are significantly upregulated in CBFB-MYH11, MMLT3-KMT2A and RUNX1-RUNX1T1 AML (**Sup. Fig. 10 A**).

Intriguingly however, increased *ZEB1* levels in AML, irrespective of driver mutations present, significantly correlated with decreased overall survival whereas increased *ZEB2* levels did not significantly alter overall survival (**Sup. Fig. 10 B**). This is despite the high overall levels of *ZEB2* in several distinct AML populations with known driver mutations such as MMLT3-KMT2A and being independently identified as an important genetic determinant in AML survival identified in human and murine AML settings (Li et al., *Blood* 2017).

We have also examined the correlation of *ZEB1* and *ZEB2* levels with the number of leukemic blasts present in AML samples and could show a significant positive correlation with leukemic blast cell numbers present (**Sup. Fig. 10 C**) that could be linked to the high expression of both *Zeb1* and *Zeb2* in HSCs that may represent the cells of origin of transformed AML populations.

Stavropoulou et al., (*Cancer Cell* 2016) have demonstrated that enhanced *Zeb1* in this leukemic stem cell population is important for AML proliferation and dissemination and may ultimately highlight where *ZEB2* and *ZEB1* expression may be most important in AML disease. These findings are highligted in the results section of the manuscript on page 19-20 and in the discussion on page 30 and highlight again why we believe that ZEB1 and ZEB2 are most likely playing oncogenic roles in AML but may be contextual.

* What is the expression of Zeb1 and Zeb2 in hematopoietic lineages? Figure 1 demonstrates stem and progenitor populations, but it is not mentioned in the manuscript more mature populations. This data should be summarized and referenced, if previously published.

This data is available from the Immgen database and we have included this data as our new Supplementary Figure 1 in the manuscript and refer to this data on page 7 on the manuscript.

* Figure 1A is very difficult to interpret, and would benefit from being displayed differently (ex; violin plot). It is unclear what the cell types indicated on the x-axis represent. What is meant by projected, broad, etc.?

We have simplified Figure 1A and have made it into a violin plot as suggested by the reviewer and simplified the cell populations and using colour-coding heatmap to highlight the percentage of cells that express Zeb1 and Zeb2 in each of the populations. The various populations analysed are defined in the Figure legend.

* In Figure 2, the authors display data for myeloid populations, however lymphoid populations are not mentioned.

We apologize for this oversight and now have included T-cell analysis from the constitutive Tie2-Cre fetal liver transplant model in **Figure 2 F** and T-cell analysis from tamoxifen-inducible *Zeb1* deletion in adult bone marrow transplant settings in **Supplementary Figure 5 D, E**. B-cell quantification using B220 analysis from the Tie2-Cre fetal liver transplant model is included in **Supplementary Figure 3 E**.

Also, the SLAM markers (CD48 and CD150) are the standard markers for HSC populations and should be performed in parallel with CD34 and CD135.

We did utilize both markers in parallel and have included our gating strategy from wild-type bone marrow for HSPC analysis in **Figure 2 B**. We have included SLAM marker analysis (CD150/CD48) to demonstrate no differences in LT-HSCs in the Zeb1 deficient reconstituted mice compared to controls in **Figure 2 C** and **Supplementary Figure 3 A**. From the review publication by Challen et al., Cytometry 2009 from the Goodell lab the use of CD34 and CD135 can be quite equivalent in defining MPP, as well as LT-and ST-HSC populations. We have decided to use this strategy throughout our manuscript given that the gating of these three populations is a little bit better defined than with CD150 and CD48 in our hands. We feel that both strategies have their strengths and weaknesses, however.

* On page 8, the authors look at CMP population, which is listed as LK CD34+ CD16/32-. This is inaccurate since the CMP population is CD16/32 low. The authors should include a gating strategy for the CMP, MEP, and GMP populations.

We wish to apologize for these typos and have better defined our HSPC gating strategy in **Figure 2 B**. In particular, we have better defined our analysis of the MPP population (Lin^-^cKit^+^Sca1^-^) by using FCgR_II/III_/CD16-CD34 staining analysis to more distinctly identify the CMP (CD34^+^FCgR_II/III_^low^), GMP (CD34^+^FCgR_II/III_^+^), and MEP (CD34^-^FCgR_II/III_^-^) populations. These changes are indicated on page 8 of the new manuscript.

* Also, in page 8 the authors say "Overall, these results are in contrast to Zeb2 deficient hematopoietic null mice that showed enhanced granulocytic differentiation, decreased GMPs…." Is this completely in contrast? In Figure 2C, the bar graph shows a decrease of GMP.

We agree and have modified the text accordingly. The new text has been adapted on page 9 to read “Overall, these results **are slightly** in contrast to *Zeb2* deficient hematopoietic null mice that showed enhanced granulocytic differentiation, as well as increased HSPC populations including MEPs that suggests **potentially** divergent roles of *Zeb1* and *Zeb2* in controlling multilineage hematopoietic differentiation (9).”

In addition, the LSK population contains the LT-HSC, ST-HSC, and MPP, however the data displayed for % live cells depicts more LT-HSC and ST-HSC than total LSK.

We wish to thank the reviewer for catching this mistake. The wrong numbers were initially used for this Lin^-^Sca^+^cKit^+^ population. We have adapted **Figure 2 D** and **Supplementary Figure 3 B** accordingly and show that the decreases in % and total numbers of this LSK population is reflected in the decreases in ST-HSC and MPPs observed. This is also highlighted in the new text on page 8.

* Figure 3C, what is the overall chimerism, and what is the representation of each lineage?

We have included the overall percentage chimerism and the percentage lineage chimerism in the new Supplementary Table I. This data has been referred to on page 10 of the manuscript.

* On page 15, the authors state "Overall, we have discovered that many genes may be co-repressed by Zeb1 and Zeb2…." RNAseq data alone does not support this conclusion. This would need validation.

Given that the LSK population that we are analysing is quite small and technically challenging to work with it is not feasible to perform ChIPseq experimental validation on this limited cell population to prove that these upregulated targets are normally bound by ZEB1 and ZEB2 and repressed by these transcription factors. We have instead employed a bioinformatics-based approach using publicly available ChIPseq data for ZEB1 and ZEB2 in human hematopoietic cell lines. Although these two cell lines (GM12878 and K562 human cell lines respectively) have different haematopoietic origins, we observed marked occupancy of both transcription factors around the gene start site for these DEGs identified in *Zeb1/2* deficient LSK cells in this manuscript, with most of the peaks spanning 0-1 kb around these promoter sites (**Supplementary Figure 8 A** and **8 B**). Moreover, ZEB1 and ZEB2 shared a substantial proportion of bound peaks and some genes contained ZEB1 and ZEB2 binding enrichment in the same genomic regions (**Supplementary Figure 8 C**). This data along with our RNA-seq data in this manuscript have led us to conclude many genes controlled by ZEB1 and ZEB2 in the mouse are also potential transcriptional targets bound by ZEB1/2 in human cells, demonstrating the existence of a conserved network controlled by ZEB1/2. This new data has been added to pages 15-16.

* For Figure 7, a number of details are missing, including time-points, age, significance, impact on survival?

5-month-old female transgenic mice were used in this analysis and this information has been added to the figure legend for this new Figure 8. Significance values have been added to all figures and in the main text throughout the manuscript. In terms of the impact of hematopoietic restricted R26 locus based *Zeb1* and *Zeb2* overexpression on survival the following paragraph was added to the top of page19.

“Of note, hematopoietic restricted expression of *Zeb2* leads to the development of ETP-ALL from 6 months of age onwards (15) but only develop AML when inter-crossed onto a p53 conditional null tumour-prone background in approximately 10% of mice analysed (15). Hematopoietic *Zeb1* overexpressing mice do not spontaneously develop T cell or myeloid malignancies up to 1.5 years of age and appear to have a normal life expectancy (48).”

Also, it would be beneficial to include protein expression along with GFP expression. Is data significant with only 3 representative mice?

Western blot analysis for ZEB1 protein expression in transgenic Vav-iCre;Zeb^tg/tg^ and Zeb2^tg/tg^ spleen and bone marrow have been added to Figure 8 D and Supplementary Figure 9A respectively. Significance values have been added to the Figure legends and main results text throughout the manuscript.

* Experimental details are lacking for Figure 8. Was only one primary donor used for the cohort?

Yes, single primary donors were used for the creation of the cohorts whereby 10,000 GFP^+^cKit^hi^CD11b^+^ primary leukemic stem cell enriched populations for each genotype were used to eliminate some variation in engraftment that is common in this type of secondary tumour engraftment experiments.

Was the phenotype of the primary mice identical?

Yes, all mice developed primary AML.

What was the tumor burden similar?

When primary and secondary recipients showed the first signs of illness, they were humanely sacrificed. Flow cytometry of bone marrow showed around 90% CD11B^+^EGFP^+^ blast count in the bone marrow indicative of high tumour burden that is common for this aggressive MLL-AF9 driven AML.

Were these leukemia cells from peripheral blood, spleen or bone marrow?

GFP^+^cKit^hi^CD11b^+^ primary leukemic cells from each genotype were isolated from the bone marrow and used to establish cohorts of secondary engrafted mice.

Minor Weaknesses:

* Manuscript needs attention to proper gene and protein nomenclature.

We have now used proper HUGO protein and gene name nomenclature throughout the manuscript.

* A number of figures that font is too small to be legible (ex; Figure 6, Figure 5C)

We have now attempted to enhance the legibility by splitting the DEG heatmaps and bioinformatics analysis into new separate **Figures 6** and **7** respectively.

* The gating strategy is unclear in Figure 2, are numbers based on gating of CD45.2+ cells?

Yes. The average percentage of CD45.2+ cells in PB was 87.1% 3 weeks after transplantation, 93.2% by 8 weeks and more than 96% by 23 weeks.

* Numbers in Figure 3A are confusing. Why in 1st plating is there 8 and 7 but in secondary 4 and 6, but at the end of the figure legend it says 4 biological replicates?

We apologize for the confusion. One issue from these long-term methylcellulose replating assays is that some cultures were lost due to contamination despite the use of antibiotics and anti-fungals in the culture media. We have now repeated these methylcellulose replating assays according to Reviewer 2’s request using the tamoxifen inducible system and adult bone marrow to demonstrate the loss of replating potential of *Zeb1* deficient HSPCs. This new data is presented in the new **Supplementary Figure 5 C** and discussed on page 11 of the manuscript.

* Figure 5 key doesn't match the text

Figure legend has been adapted accordingly to match the text.

**Rev. 2:**
In this manuscript, the authors investigate the role of Zeb1 and Zeb2 in hematopoiesis, using conditional mouse knockout and overexpression models. A number of other investigators have published related articles, and therefore the authors focused on the effects of Zeb1 and double Zeb1/Zeb2 knockouts. The provide data on Zeb1 and Zeb2 expression, the effects of deletion, rescue by Zeb2, assessment of effects on downstream genes following deletion, overexpression, and effect of loss of Zeb1/2 on an AML model. In general, the experiments complement studies from other laboratories, and add to our knowledge of the role of Zeb1/2 in hematopoiesis and leukemia. This reviewer has a number of suggestions for improvement of the manuscript.

(1) In general, the authors utilize fetal liver cells rather than adult bone marrow in analyzing the effects of loss of Zeb1, perhaps because of the study of Almotiri et al. They should discuss how use of fetal versus adult cells may or may not lead to differences, given that fetal HSCs are proliferative and adult relatively non-proliferative. Can this account for any difference with the study of Almotiri?

There very well may be several reasons for the differences observed between our study and the one published by Almotiri. In the first instance this group has used the MX1 interferon inducible Cre line without transplant in their initial hematopoietic analysis. As the MX1 cre line is not 100% specific for the hematopoietic lineage it is hard to interpret some of their data as there may be stromal effects as well as cell autonomous effects associated with *Zeb1* deletion. Therefore, we initially used hematopoietic enhanced or restricted lines such as the Tie2 and Vav-iCre lines respectively in combination with hematopoietic reconstitution to address the cell autonomous role of *Zeb1* deletion in hematopoiesis. In addition, the use of poly:IC can generate an inflammatory interferon response that may have subtle effects on HSC quiescence causing them to proliferate (Sato et al, *Nature Medicine*, 2009).

(2) Similarly, the authors should discuss potential differences between fetal liver cell transplants into adult recipients (Fig. 2) versus using adult bone marrow as donor.

Fetal liver (FL) derived HSCs are more highly proliferative than bone marrow (BM) derived adult HSCs and appear to utilize oxidative metabolic pathways more than BM HSCs and may be better protected from ROS-mediated genotoxicity (Manesia el al., *Stem Cell Res* 2015). As well, in human fetal and adult HSCs there have been differences demonstrated in their ability to give rise to distinct T cell lineages in humans (Mold et al., *Science* 2010). Given that we do see differences in T-cell differentiation of *Zeb1* deficient HSPCs in our study and the one published by Almotiri et al., we have added these aspects to the discussion on page 29 of the discussion along with the above aspects concerning the use of MX1-Cre to explain some of the subtle differences between the two studies.

(3) The replating assays (Fig. 3A) were performed with fetal liver, and only for 2 replatings.

The reason why we stopped after two replatings is that the *Zeb1* deficient cultures were already showing significant decreases and very low number of colony formation at this time if any.

If the authors have to analyze fetal and liver and/or adult bone marrow from Zeb1 KO mice for other experiments, they should repeat the fetal liver and compare with adult bone marrow (continuing, for at least the adult, for 3-4 rounds. However, this reviewer would not insist on breeding mice solely for this purpose.

We have repeated these experiments using the tamoxifen-inducible deletion of *Zeb1* and adult bone marrow and have demonstrated that here as well Zeb1 deletion results in decreased replating potential of *Zeb1* deficient HSPCs and that by the second replating there are very few colonies if any emerging compared to Cre only controls (**Supplementary Figure 5 C**, **p<0.01).

(4) While the authors are utilizing mouse strains described in previous publications, this reviewer would like to see more information about the knockouts without having to look up a another paper. It is suggested to provide a diagram and some information about the Zeb1 and Zeb2 knockout strategy.

We have added a diagram in **Figure 4 A** (on the right) to highlight the conditional loss of function *Zeb1* and *Zeb2* alleles used in this study as well as the structure of these alleles after Cre-mediated excision of exon 6 and 7 respectively in the two models. The diagram of the conditional R26-*Zeb1* gain of function allele is depicted in **Figure 8 A** and the R26-*Zeb2* allele in **Supplementary Figure 9** is essentially the same as the Zeb1 allele depicted in **Figure 8 A** except that it is Zeb2 and not Zeb1 as cDNA downstream of the floxed STOP cassette.

In addition, the authors should provide information in the supplement showing loss of RNA and protein in the knockouts. Was any residual non-deleted RNA or protein detected?

To begin to address this issue, we looked at DNA isolated from the whole bone marrow by PCR analysis in the tamoxifen-inducible model 2 weeks after engraftment. Here, we could see some evidence of the presence of continued residual floxed allele along with the Cre-excised allele. As well, we could detect the presence of wild-type allele most probably from the stromal compartment of the recipient wild type mouse. However, despite the continued presence of floxed allele we could demonstrate clear phenotypes associated with loss of *Zeb1* in the replating assays and T-cell development in reconstituted mice compared to Cre only controls (**Supplementary Figure 5 C-E**).

(5) A number of investigators have demonstrated data in both murine and human models that some degree of myeloid differentiation is essential for leukemic transformation in AML. The authors should discuss whether this might have some relationship to the enhanced survival observed in the MLL-AF9 model?

This is an excellent point raised by the reviewer and in primary AML disease development, some degree of myeloid differentiation is required for transformation (see Ye et al, *Cell Stem Cell*, 2015). In our MLL-AF9 model we are deleting *Zeb2* or both *Zeb1/2* after established disease in secondary transplant settings. Given that HSC deficiency in both *Zeb1* and *Zeb2* have such a strong differentiation block it would be predicted that deletion of both *Zeb1* and *Zeb2* in HSCs prior to MLL-AF9 transduction would have dramatic effects on primary AML development that are more severe than in secondary established AML disease. We are presently investigating this possibility and will form the basis of a separate study. This issue has now been added to the discussion on page 27.

__________________________________________________
In compliance with data protection regulations, you may request that we remove your personal registration details at any time.  (Use the following URL: <https://www.editorialmanager.com/pbiology/login.asp?a=r>). Please contact the publication office if you have any questions.
