# Supplementary material for: Interplay between the EMT transcription factors ZEB1 and ZEB2 regulates hematopoietic stem and progenitor cell differentiation and hematopoietic lineage fidelity
Source: PLoS Biol. 2021 Sep 22;19(9):e3001394. doi: 10.1371/journal.pbio.3001394 (PMC8489726; doi:10.1371/journal.pbio.3001394)
Supplement: Attachment — Submitted filename: Wang et al., Response to reviewers comments-R2.docx [file pbio.3001394.s023.docx]

We would like to start out by thanking the reviewers for their additional positive comments and suggestions. Wherever possible we have tried to address their comments/concerns either experimentally or through further bioinformatics analysis. Changes in the manuscript are highlighted in red in the manuscript and our response to the reviewer’s comments are highlighted in blue below.

Reviewers' comments

Rev. 1:
Accept

**Thank you**


Rev. 2:
   In general, the authors have done a very good job addressing the reviewer's comments. This reviewer has only two comments:

    One comment was the suggestion to use proper gene and protein nomenclature, which the authors proceeded to do in the revised manuscript. Although the authors have to do this to satisfy the "Nomenclature Police", this reviewer would like to make the point that HUGO rules can sometimes be very useful (as in the case of RUNX family nomenclature), and sometimes be completely idiotic (do you know what spfi1 is? Is it PU.1 or SP1, they are VERY DIFFERENT transcription factors, and HUGO nomenclature is causing great confusion, not clarification!). We scientists should not have to be slaves to people who are ignorant of what they are doing, but unfortunately we are. When this reviewer is Emperor, this will change.

**I will vote for this.**

    A second comment was "In addition, the authors should provide information in the supplement showing loss of RNA and protein in the knockouts. Was any residual non-deleted RNA or protein detected?" The authors commented on a residual floxed allele, which represents DNA, not RNA or protein. If the authors have RNA and/or protein data available, it would be good to include it as part of a supplementary figure, but this is a relatively minor point and this reviewer would not insist on additional experimentation for this.

**We have used a bioinformatics approach and the original RNA-seq data obtained from LSK cells after 4 days of tamoxifen-mediated deletion of Zeb1 and Zeb2 or both alleles. Using this analysis, we could demonstrate an approximate 60% deletion of the floxed Exon 6 of *Zeb1* in both *Zeb1* single and *Zeb1/2* DKO settings and an approximate 90% deletion of Exon 7 from the floxed *Zeb2* allele in single *Zeb2* and *Zeb1/2* DKO settings. This new data has been added as a new Supplementary Figure 7 and on page 14 of the results section of the manuscript and raw data included in Supplementary Table 6.**
